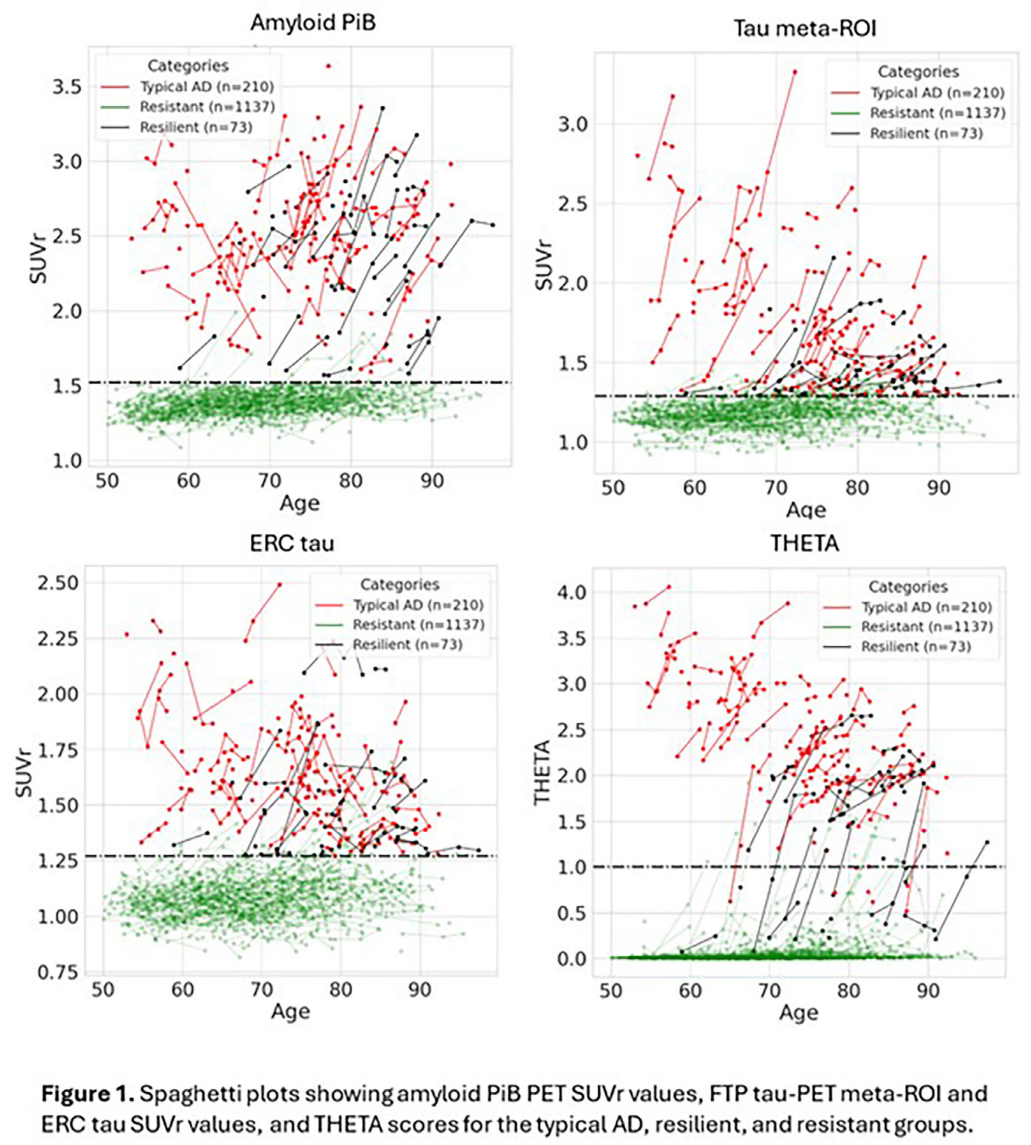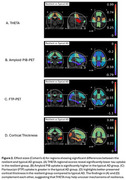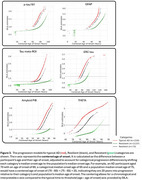# Precise Estimation Of Heterogenous Global Tau Burden In The Brain Sheds Light On Cognitive Resilience Mechanisms

**DOI:** 10.1002/alz70856_103992

**Published:** 2025-12-26

**Authors:** Robel K Gebre, Scott A. Przybelski, Sheelakumari Raghavan, Jeffrey L. Gunter, Val J Lowe, Jonathan Graff‐Radford, David S. Knopman, Ronald Petersen, Clifford R. Jack, Prashanthi Vemuri

**Affiliations:** ^1^ Mayo Clinic, Rochester, MN, USA; ^2^ Department of Neurology, Mayo Clinic, Rochester, MN, USA

## Abstract

**Background:**

Cognitive resilience to Alzheimer's disease (AD) pathology has been widely studied, but distinguishing resilient individuals from those with typical tau pathologies remains challenging due to the overlap in imaging and plasma biomarker profiles. We hypothesize that our machine learning‐based (ML) tau heterogeneity score, THETA (JNUMED, July 2024), could serve as an effective marker of resilience and provide a better estimation of tau's temporal progression.

**Method:**

Longitudinal imaging data from the Mayo Clinic Study of Aging (MCSA, *n* = 1834) and ADRC (*n* = 246) were analyzed. Participants were categorized into typical AD and resilient groups using amyloid PiB‐PET (abnormal PiB‐PET≥25 Centiloid units), Flortaucipir (FTP‐PET) (meta‐ROI≥1.29 SUVr, ERC≥1.27 SUVr) and Mini‐Mental State Examination (MMSE). Typical AD was defined as abnormal PiB‐PET and abnormal FTP‐PET for both biomarkers (Figure 1). Resilience to AD pathology was determined by predicted values of MMSE >28 from a linear mixed effects model for cognitively unimpaired participants. In addition, a resistant group was broadly defined as having normal PiB‐PET. To capture the spatial heterogeneity of tau distribution in the brain, regional THETA scores were generated using ML model and visual ratings. Additionally, a progression model, Sampled Iterative Local Approximation (SILA), was fitted using AD and plasma biomarkers (*p*‐tau181 and GFAP) for validation.

**Result:**

THETA identified regions of reduced tau uptake in the frontal, parietal, temporal, occipital, and limbic regions when compared to the typical AD group (Figure 2A). This was confirmed by the preserved cortical thickness in these same regions (Figure 2D). In addition, SILA showed THETA can distinguish global tau temporal progressions of typical AD, resilient, and resistant groups compared to the other biomarkers (Figure 3). Interestingly, the onset age for GFAP was younger for resilient and typical AD, indicating earlier onset of inflammation in the presence of pathology.

**Conclusion:**

The significant regions identified by THETA, showing reduced tau and preserved cortical thickness, combined with the clear offset in progression models between resilient and typical AD, suggest that THETA may help uncover pathological tau mechanisms that differentiate these groups. These findings highlight the potential of THETA to provide valuable insights into resilience mechanisms and enhance our understanding of AD.